# Early and Medium Outcomes of On-Pump Beating-Heart *versus* Off-Pump CABG in Patients with Moderate Left Ventricular Dysfunction

**DOI:** 10.21470/1678-9741-2018-0207

**Published:** 2019

**Authors:** Weitie Wang, Yong Wang, Hulin Piao, Bo Li, Tiance Wang, Dan Li, Zhicheng Zhu, Rihao Xu, Kexiang Liu

**Affiliations:** 1 Department of Cardiovascular Surgery, 2nd Hospital of Bethune, Jilin University, Changchun, Jilin, China.

**Keywords:** Coronary Artery Bypass, Off-Pump, Ventricular Dysfunction, Left, Coronary Artery Bypass, Myocardial Revascularization

## Abstract

**Objective:**

This study aims to compare the early and medium outcomes of on-pump beating-heart (OPBH) coronary artery bypass grafting (CABG) and off-pump CABG (OPCABG) in patients with left ventricular ejection fraction (LVEF) between 30% and 40%.

**Methods:**

This is a retrospective study of ischemic heart disease patients with LVEF between 30% and 40% who underwent surgical revascularization from January 2013 to December 2017. Patients were divided into OPBH group (n=44) and OPCABG group (n=68), according to the surgical method. Clinical material with early and medium outcomes were investigated and compared between these groups.

**Results:**

The two groups had similar baseline. Two OPBH patients and 3 OPCABG patients died in the hospital, which had no statistical significance (*P*>0.05). OPBH patients received a greater number of grafts (3.74±0.84) and presented more improved LVEF (45.92±7.11%) than OPCABG patients (3.36±0.80) and (42.81±9.29%), respectively, which had statistical significance (*P*<0.05). An increased amount of drainage during the first 12 hours was found in the OPBH group (*P*<0.05). Reoperation for bleeding, duration of mechanic ventilation, and other early outcomes had no statistical significance between the two groups. During the medium-time follow-up, OPBH patients showed significantly lower major adverse cardiovascular events (MACE)-free survival time (*P*=0.049) than OPCABG patients.

**Conclusion:**

The OPBH technique was a safe and an acceptable alternative for surgical revascularization in patients with moderate left ventricular dysfunction which provided better mid-term MACE-free survival compared with OPCABG.

**Table:** 

Abbreviations, acronyms & symbols				
ACT	= Activated clotting time		LCOS	= Low cardiac output symptom
AKI	= Acute kidney injury		LITA	= Left internal thoracic artery
BMI	= Body mass index		LVEDD	= Left ventricular end-diastolic dimension
CABG	= Coronary artery bypass grafting		LVEF	= Left ventricular ejection fraction
CAD	= Coronary artery disease		MACE	= Major adverse cardiovascular events
CCF	= Congestive cardiac failure		MI	= Myocardial infarction
COPD	= Chronic obstructive pulmonary disease		NYHA	= New York Heart Association
CPB	= Cardiopulmonary bypass		OPBH	= On-pump beating-heart
CRD	= Chronic renal dysfunction		OPCABG	= Off-pump CABG
CRF	= Chronic renal failure		PCI	= Percutaneous coronary intervention
DM	= Diabetes mellitus		RITA	= Right internal thoracic artery
DSWI	= Deep surgical sternal wound infection		SD	= Standard deviation
ECG	= Electrocardiogram		STS	= Society of Thoracic Surgeons
EF	= Ejection fraction		SVG	= Saphenous vein graft
GFR	= General regulatory factor		TEE	= Transesophageal echocardiography
IABP	= Intra-aortic balloon pump		TTE	= Transthoracic echocardiography
ICU	= Intensive care unit			

## INTRODUCTION

Conventional coronary artery bypass grafting (CABG) had been used to surgical revascularization of patients with ischemic heart disease for many years. However, the use of cardiopulmonary bypass (CPB) and cardioplegic arrest might contribute to some related complications. Nowadays, off-pump CABG (OPCABG) has been gradually used to reduce the intraoperative and postoperative complications related to CPB and cardioplegic arrest, especially in some high-risk patients^[1,2]^. Initially, it seemed a suitable strategy for surgical revascularization of most patients. Unfortunately, transient hemodynamic instability would occur during the heart displacement maneuvers and extensive surgical manipulations which would lead to urgent conversion and cause some severe adverse results. In addition, OPCABG was always reported with incomplete revascularization^[3]^ and emergence intraoperative conversion^[4,5]^ caused by hemodynamic deterioration, especially in patients with severe left ventricular dysfunction, which increased the morbidity and mortality during operation.

So a new hybrid method, the on-pump beating-heart^[6]^ (OPBH) CABG, has been used in these high-risk patients in recent years^[7,8]^. This technique could maintain the coronary blood flow to reduce myocardial injury^[9]^ and reduce the preload and afterload to decrease myocardial oxygen demand. It was a compromise choice which ensured intraoperative hemodynamic stability and avoided complications caused by aortic cross-clamp and cardioplegic arrest. In addition, it had been demonstrated that the beating heart during the operation could also reduce myocardial edema and inflammatory response^[9]^.

So whether this hybrid method was the best strategy for the patients, especially those with a left ventricular ejection fraction (LVEF) between 30% to 40%, it remained controversial^[10,11]^. This study aimed to evaluate the early and medium outcomes of OPCABG and OPBH CABG.

## METHODS

This study was conducted as a retrospective observation from January 2013 to December 2017 and was approved by the Jilin University's Ethics Committee. All operations were performed by the same surgeons. A total of 1152 patients went to the Department of Cardiovascular Surgery of the 2^nd^ Hospital of Bethune of Jilin University for surgical revascularization during this period; 858 patients underwent OPCABG surgery and 294 patients underwent CABG surgery with CPB assist. Inclusion criteria comprised patients with: 1, an ejection fraction (EF) value evaluated by pre-operation transthoracic echocardiography (TTE) between 30% and 40%; 2, ischemic heart disease that met surgical revascularization criteria; 3, no other cardiac disease, such as ventricular septal defect, medium to severe mitral regurgitation, and left ventricle aneurysm, that needed to be intervened at the same time. Finally, 998 patients with LVEF above 40% and 42 patients with other cardiac disease treated at the same time were excluded and 112 patients were selected for this study. All patients were divided into 2 groups, according to the revascularization method: OPCABG group and OPBH group.

The study design was approved by the Jilin University's Clinical Trial Ethics Committee and consent was obtained for publication.

### Study Population

There were 31 (70.45%) males in the OPBH group and 44 (70.58%) males in the OPCABG group. The mean age was 60.48±9.44 and 61.22±9.59 years old, respectively, in the OPBH and the OPCABG group. More detailed baseline characteristics are shown in Table 1. There were no significant differences in age, gender, obesity, smoking, New York Heart Association (NYHA) class III-IV, previous myocardial infarction (MI), previous percutaneous coronary intervention (PCI), hypertension, diabetes mellitus (DM), chronic renal failure (CRF), recent MI, congestive heart failure, hyperlipemia, chronic obstructive pulmonary disease (COPD), prior cerebrovascular accident, abnormal motion of the segmental cardiac wall, LVEF, left ventricular end-diastolic dimension (LVEDD), extent of coronary artery disease (CAD), and EuroSCORE between the 2 groups.

**Table 1:** Baseline and procedural characteristics after matching.

		OPBH group(n=44)	OPCABG group(n=68)	*P* value
Age (years old)		60.48±9.44	61.22±9.59	0.689
Older age (>65 years old)		10 (22.73%)	16 (23.52%)	0.922
Male		31 (70.45%)	48 (70.58%)	0.988
Obesity (BMI >30 kg/m^2^)		25 (56.82%)	40 (58.82%)	0.834
Smoking		21 (47.73%)	35 (51.47%)	0.699
NYHA class III-IV		35 (79.54%)	52 (76.47%)	0.703
Previous MI		29 (65.91%)	43 (63.23%)	0.773
Previous PCI		5 (11.36%)	8 (11.76%)	0.948
Hypertension		28 (63.64%)	42 (61.76%)	0.842
Diabetes mellitus		7 (15.91%)	12 (17.64%)	0.811
Chronic renal dysfunction		2 (4.54%)	3 (4.41%)	0.973
Recent MI		6 (13.64%)	10 (14.71%)	0.875
Congestive heart failure		10 (22.73%)	15 (22.06%)	0.934
Hyperlipemia		32 (72.73%)	49 (72.06%)	0.938
COPD		5 (11.36%)	9 (13.24%)	0.770
Prior cerebrovascular accident		28 (63.64%)	44 (64.71%)	0.908
Abnormal motion of the segmental cardiac wall		24 (54.55%)	38 (55.88%)	0.890
LVEF		34.92±4.49	34.41±4.55	0.562
LVEDD		62.11±6.31	60.45±6.91	0.202
Enlarged left ventricles (LVEDD >65 mm)		12 (27.27%)	16 (23.53%)	0.655
Extent of CAD	Left main stem disease	11(25.00%)	18 (26.47%)	0.862
	Three vessels	40 (90.91%)	62 (91.18%)	0.961
	Two vessels	3 (7.50%)	5 (7.35%)	0.915
	Logistic EuroSCORE	7.52±2.71	7.68±2.99	0.775

BMI=body mass index; CAD=coronary artery disease; COPD=chronic obstructive pulmonary disease; LVEDD=left ventricular end-diastolic dimension; LVEF=left ventricular ejection fraction; MI=myocardial infarction; NYHA=New York Heart Association; OPBH=on-pump beating-heart; OPCABG=off-pump coronary artery bypass grafting; PCI=percutaneous coronary intervention

### Surgical Procedures

Intraoperative transesophageal echocardiography (TEE) was introduced into the esophagus after general anesthesia. The surgery was operated through a median full sternotomy. Left and right internal thoracic arteries (LITA and RITA) and saphenous vein grafts (SVG) were harvested at the same time using "no-touch" technique. Deep pericardial sutures were performed after incision of the pericardium. Surgical revascularization was always started from the LITA going to the left anterior descending coronary territory. Then a sequential technique, right coronary, left circumflex and diagonal, was followed using one SVG. Side-to-side anti-parallel anastomoses were performed with Prolene 7-0 for the sequential bypasses. End-to-side anastomoses were performed with Prolene 6-0 for the proximal aortic connections with the saphenous vein. The anastomosis quality was assessed by transit-time flow probe (Medistim Butterfly Flow Meter, Oslo, Norway). All the target vessels were exposed and controlled with silastic sling. A CO_2_-blower mister device was used to visualize the operative field. After the anastomosis, heparin was neutralised with protamine to return the activated clotting time (ACT) to the preoperative level. A cell salvage device was used during surgery and the salvaged blood was reinfused into the patient after bleeding.

In OPBH CABG patients: systemic heparinization was implemented by 3 mg/kg and after the ACT was longer than 480 seconds, CPB was established through ascending aorta and right atrium without cardioplegic arrest or an aortic cross-clamp. Stabilization devices were the Medtronic Octopus apical suction positioning device and the Starfish apical suction positioning device (Medtronic, Inc., Minneapolis, MN, USA). The patients' systemic temperature was approximately 36ºC.

In OPCABG patients: heparin was implemented by 1 mg/kg. Stabilization devices were the Medtronic Octopus apical suction positioning device and the Starfish apical suction positioning device (Medtronic, Inc., Minneapolis, MN, USA). To avoid hypothermia-induced arrhythmia, central temperature was maintained above 36ºC.

Baseline clinical data included age, sex, obesity, smoking, NYHA class, PCI, DM, chronic renal dysfunction (CRD), previous MI, recent MI, congestive heart failure, hypertension, hyperlipemia, COPD, stroke, prior cerebrovascular accident, abnormal motion of the segmental cardiac wall, LVEF, LVEDD, enlarged left ventricles, anatomical severity of CAD, and EuroSCORE. Operative data included operation time, number of distal anastomosis, CPB time, SVG, LITA and RITA use, composite graft, urgent switch to on-pump, and prophylactic intra-aortic balloon pump (IABP) support. Bivariate analyses were used to examine differences in baseline characteristics between the two groups.

The primary endpoints studied overall death, including in-hospital mortality and death after 30 days or after discharge. The secondary endpoints were the mid-term death and the major adverse cardiovascular events (MACE) rates, such as low cardiac output symptom (LCOS), the need for repeated revascularization, new onset of acute MI, and other cardiac-related complications. Follow-up information was obtained by visit or telephone calls and was agreed by all patients before discharge by informed consent. All surviving patients underwent postoperative echocardiographic re-examination. The mean follow-up time was 38.94±16.73 (6-67) months.

### Definitions

Surgical mortality = death occurring in hospitalization and within 30 days of the procedure; Resternotomy for bleeding = reoperation to control bleeding within 36 hours following initial surgery; Postoperative MI = the appearance of new Q waves in 2 or more contiguous leads on the electrocardiogram (ECG); Postoperative LCOS = requirement for IABP and/or inotropic support for more than 30 min; Atrial/ventricular arrhythmia after OPCABG surgery = any episode of atrial/ventricular fibrillation that was registered by the monitoring system on a rhythm strip or the 12-lead ECG; Postoperative respiratory failure = duration of mechanical ventilation for more than 72 hours or reintubation following surgery; Postoperative pneumonia = a positive result in a sputum culture requiring anti-infective treatment, or chest X-ray diagnosis of pneumonia following cardiac surgery; Stroke = new permanent neurological event lasting over than 24 h; Deep sternal wound infection = bone related; any drainage of purulent material from the sternotomy wound and instability of the sternum; Acute kidney injury (AKI) = defined and classified according to the criteria proposed by the Acute Kidney Injury Network; CRF = patients whose general regulatory factor (GFR) declines to 15-20 ml/min with severe symptoms related to uraemia that can be relieved only by renal replacement therapy; Emergency conversion = the use of CPB during OPCABG due to cardiac arrest, hemodynamic compromise, ischemic episodes, and hemorrhage.

### Statistics

Continuous data were expressed as mean ± standard deviation (SD), categorical variables were expressed as numbers (percentages). Normally and non-normally distributed continuous variables were compared using Student *t*-test and Mann-Whitney U test, respectively. The Fisher's exact test or the chi-square test was used to compare categorical variables. Cumulative survival curves for long-term MACE were constructed using the Kaplan-Meier method, whereas differences between the groups were evaluated with log-rank tests. *P* value less than 0.05 was considered statistically significant. All statistical analyses were carried out by the software SPSS 19.0.

## RESULTS

### Intraoperative Data

Operation time was 261±49 min in the OPBH group and 223±55 min in the OPCABG group (*P*<0.0003). CPB time was 51.8±20.6 min in the OPBH group. The number of distal anastomosis ranged from 2 to 6 and it was 3.74±0.84 in the OPBH group and 3.36±0.80 in the OPCABG group (*P*<0.0001). The internal thoracic artery was used in all patients and SVG number was 43 in the OPBH group and 67 in the OPCABG group. Five (7.35%) patients in the OPCABG group experienced urgent switch to OPBH CABG surgery due to hemodynamic deterioration (2) or ventricular fibrillation (3) during OPCABG operation. Prophylactic IABP support was used in 4 (9.09%) patients in the OPBH group and 9 (13.23%) in the OPCABG group (*P*=0.504). More detailed data are shown in Table 2.

**Table 2:** Intraoperative data after matching.

	OPBH group (n=44)	OPCABG group (n=68)	*P* value
Operation time (minutes)	261±49	223±55	0.0003
Number of distal anastomosis	3.74±0.84	3.36±0.80	<0.0001
SVG use	43 (97.73%)	67 (98.53%)	0.754
LITA use	42 (95.45%)	66 (97.06%)	0.655
RITA use	2 (4.55%)	2 (2.94%)	0.655
Composite grafting	43 (97.73%)	67 (98.53%)	0.754
Prophylactic IABP support	4 (9.09%)	9 (13.23%)	0.504

IABP=intra-aortic balloon pump; LITA=left internal thoracic artery; OPBH=on-pump beating-heart; OPCABG=off-pump coronary artery bypass grafting; RITA=right internal thoracic artery; SVG=saphenous vein graft

Two patients (1 ventricular arrhythmia and 1 low-output syndrome) died in the OPBH group and 3 patients (2 low-output syndrome and 1 ventricular arrhythmia) died in the OPCABG group, which had no significant difference between both groups. Additionally, 2 patients died of low cardiac output (1) and malignant arrhythmia (1) among the 5 urgent switching patients in the OPCABG group. Drainage during the first 12 hours in the OPBH group (437±121 ml) was larger than in the OPCABG group (377±151 ml), which was statistically significant. There was no significant resternotomy for bleeding, duration of mechanic ventilation, intensive care unit (ICU) stay, hospital stay, ventricular arrhythmia, low-output syndrome, MI, atrial fibrillation, respiratory failure, pneumonia, and deep surgical sternal wound.

LVEF before discharge significantly improved from 34.92±4.49 to 45.92±7.11 in the OPBH group and from 34.41±4.55 to 42.81±9.29 in the OPCABG group (*P*<0.001). All survival patients have been echocardiographically examined after 6 months which showed a significant higher early postoperative LVEF in the OPBH group than in the OPCABG group (47.17±6.23 *versus* 44.52±7.01%, respectively; *P*=0.034) (Table 3).

**Table 3:** Postoperative data.

	OPBH group (n=44)	OPCABG group (n=68)	*P* value
Surgical mortality	2 (4.55%)	3 (4.41%)	0.973
Resternotomy for bleeding	__	__	__
ICU stay (days)	2.99±0.57	3.27±1.04	0.105
Hospital stay (days)	8.95±1.50	9.27±2.01	0.368
Ventricular arrhythmia	3 (6.82%)	5 (7.35%)	0.915
Low-output syndrome	1 (2.27%)	3 (4.41%)	0.551
Drainage during the first 12 hours (ml)	437±121	377±151	0.029
Stroke	2 (4.55%)	4 (5.88%)	0.759
Myocardial infarction	1 (2.27%)	2 (2.94%)	0.831
Atrial fibrillation	16 (36.36%)	28 (41.18%)	0.611
AKI requiring dialysis	2 (4.55%)	6 (8.23%)	0.391
Respiratory failure	__	1 (1.47%)	0.419
Pneumonia	2 (4.55%)	4 (6.06%)	0.784
DSWI	1 (2.27%)	2 (2.94%)	0.831
LVEF before discharge	45.92±7.11	42.81±8.29	0.043
LVEF after 6 months	47.17±6.23	44.52±7.01	0.043

AKI=acute kidney injury; DSWI=deep surgical sternal wound infection; ICU=intensive care unit; LVEF=left ventricular ejection fraction; OPBH=on-pump beating-heart; OPCABG=off-pump coronary artery bypass grafting

**Fig. 1:**
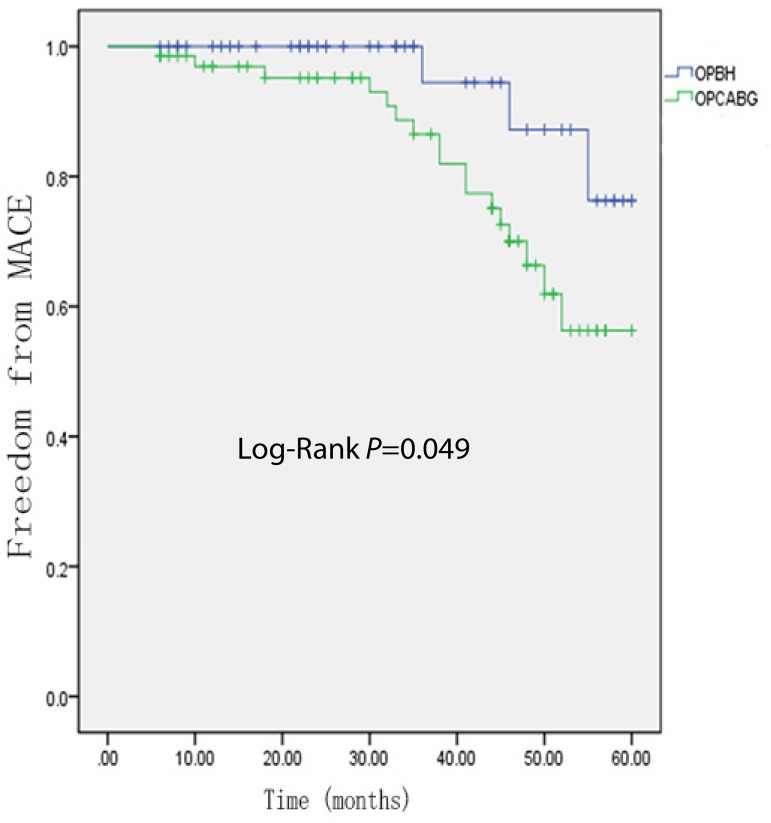
Kaplan-Meier curve estimates in the propensity-matched populations. Freedom from major adverse cardiovascular events (MACE). OPBH=on-pump beating-heart; OPCABG=off-pump coronary artery bypass grafting

**Fig. 2:**
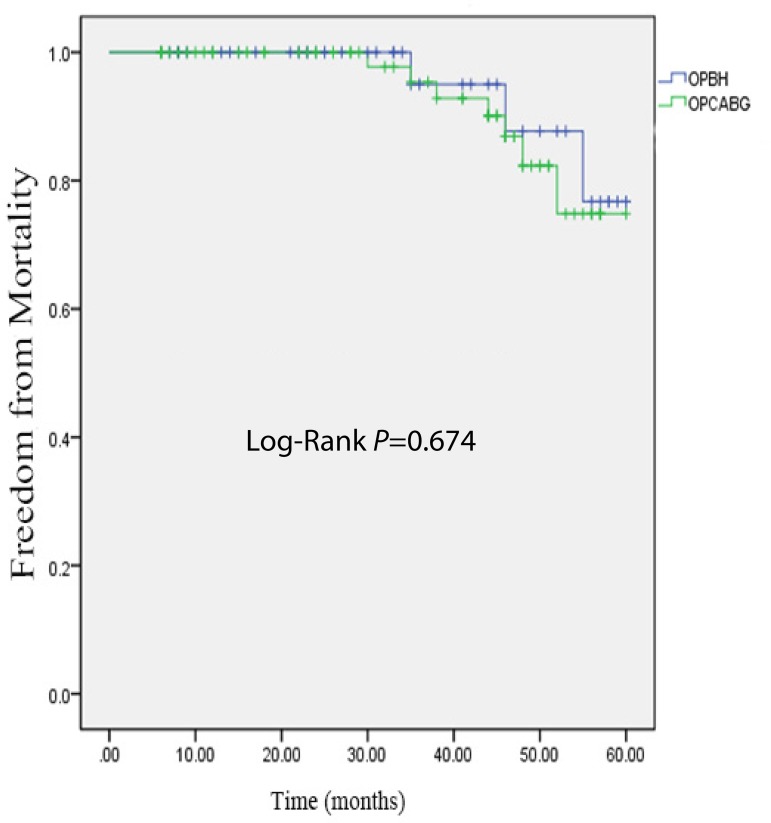
Kaplan-Meier curve estimates in the propensity-matched populations. Freedom from mortality. OPBH=on-pump beatingheart; OPCABG=off-pump coronary artery bypass grafting

All patients were followed up; the mean follow-up time was 38.94±16.73 months. Kaplan-Meier analysis of freedom from MACE revealed significantly lower event-free survival rates in the OPBH group (*P*=0.049) than in the OPCABG group (Figure 1). Kaplan-Meier analysis of freedom from mortality revealed no significant difference between both groups (*P*=0.674) (Figure 2).

## DISCUSSION

CABG had been used worldwide for surgical revascularization to treat patients with CAD for a long time. Nowadays, OPCABG has been preferred by some experienced surgeons to avoid the disadvantages caused by CPB and aorta clamp. However, considering the short-time morbidity and mortality and the long-time outcomes, the best treatment still remains in debate.

Some large randomized trials (ROOBY and CORONARY) had reported the long-time outcomes of off-pump and on-pump CABG^[12]^. But only a small number of patients with left ventricular dysfunction were included in these trials. So, for patients with moderate left ventricular dysfunction (30%<EF<40%), the best method of surgical revascularization remains controversial. Reports had shown that manipulation and hemodynamic deterioration during OPCABG might entail urgent conversion to conventional CPB (2.2-15.6%), especially in patients with left ventricular dysfunction^[13,14]^, which leads to poor prognosis and highly increased in-hospital mortality. The conversion rate had relevance, with many risk factors, such as less experienced surgeons^[15]^, patients' risk factors, and lack of technical advantages. According to a meta-analysis of recent reports, patients presented with high risk, such as poor cardiac function^[16]^, poor-quality coronary targets^[17]^, prior MI, redo or salvage revascularization^[18]^, or COPD^[19]^, accounted for the major part of conversion and their presence was predictive of a several-fold increase in the risk of conversion. Accordingly, OPBH CABG surgery in patients with moderate left ventricular dysfunction could avoid the urgent conversion during OPCABG.

Some critics might argue that low EF patients treated with off-pump technique would undergo incomplete revascularization because during OPCABG, low blood pressure and arrhythmia always appeared, especially if the position changed during circumflex anastomosis^[20]^. Some surgeons would give up continuing revascularization to avoid ventricular arrhythmia. Although incomplete revascularization would not increase early risk, the long-term outcomes, such as recurrent angina and other ischemia symptoms, decreased the late survival rate and required reintervention.

However, comparing with the incomplete revascularization caused by instable hemodynamics, the most drawback of OPCABG was urgent conversion during manipulation of the heart, which had been associated with poor prognosis and might increase many complications and in-hospital mortality. The Society of Thoracic Surgeons (STS) database showed the conversion rate (5.2%) for patients with low LVEF. Other reports showed higher conversion rates. And if the urgent conversion occurred, mortality rate would increase by more than 7 times. A study reported that the rate of in-hospital mortality ranged from 5.4% to 32.1% in emergency conversion compared with 0 to 3.6% in OPCABG patients. Several studies had used regression analysis to isolate specific predictors of conversion. The first and second specific predictors were LVEF (45.398) and congestive cardiac failure (CCF) (47.145)^[17,21]^. In addition, perioperative cardiac dysfunction might occur in more than 20% of cardiac surgery patients^[22]^. So, preventing and dealing with the resultant hemodynamic insult were central to prevent conversion.

Accordingly, for patients with normal EF, on-pump or off-pump CABG would not influence the operative mortality and long-term survival^[23]^. But for patients with poor EF, an ideal treatment is not only safe and easy during the manipulation, but also avoids the complications caused by aortic cross-clamping with CPB^[24]^. CPB circuit acting as mechanical support seemed to be safer and allowed optimal exposure of the coronary arteries, especially during revascularization of the circumflex branch to reach complete revascularization. It acted as a hybrid procedure and might be preferred in patients with low EF. Early studies had shown that the OPBH technique was safe and had satisfactory short-term clinical outcomes compared with conventional CABG. Less myocardial injury would also be observed after OPBH CABG^[9]^. So, for patients with moderate left ventricular dysfunction, our study showed that the OPBH CABG technique was effective for revascularization and myocardial functions and was associated with low postoperative morbidity and mortality, which was consistent with the findings reported in several studies.

We have found some data that agree with the outcomes of our study, such as that more drainage on the first 12 hours and a greater number of grafts significantly improved LVEF. More drainage on the first 12 hours in OPBH CABG may be related to systemic heparinization and CPB. However, the incidence of reoperation for bleeding between the 2 groups showed no significance. With the support of CPB and stabilization apparatus, more grafts were found in the OPBH group than in the OPCABG group, which were related to complete vascularization. The greater number of grafts in the OPBH group did not show more benefit in mortality rate at the early follow-up time. However, during the long-time follow-up, the Kaplan-Meier analysis of freedom from MACE showed a significantly better MACE-free period, which proved that patients with moderate left ventricular dysfunction that underwent OPBH CABG had better outcomes during the mid-time follow-up. However, the mortality incidence rate between both groups showed no significance, maybe due to the smaller sample size and shorter follow-up time. In addition, more improved LVEF in the OPBH group might be related to complete revascularization, hemodynamic stability, and less myocardial damage during operation. Although we used silastic sling instead of intracoronary shunts to expose the target vessels, which may cause ischemia, the anastomosis time of each target vessel was less than 6 min and a temporary brief coronary occlusion maneuver (less than 14 min) during anastomosis was tolerable in most of the target coronary arteries^[25]^. In addition, we used TEE to monitor the wall motion abnormalities during the coronary occlusion maneuver and we found no significant change during this period.

Most authors had shown that OPBH CABG presented a low risk for systemic hypoperfusion during surgery, which could protect the visceral organs from hypoperfusion injuries and avoid the complications caused by hemodynamic dysfunction or collapse. The kidney might be more sensitive to hypoperfusion^[26]^. Our study showed a slightly lower incidence of AKI requiring dialysis in the OPBH group than in the OPCABG group, although there was no significant difference. The stroke also had a slightly lower incidence because of stable hemodynamic and no aortic cross-clamping.

Our study also showed that the left ventricle's size in the OPBH group was slightly larger than in the OPCABG group. The larger size of the left ventricle maybe more hypertrophied, stiff, and made the leftward displaced hearts difficult to position optimally. This anatomical disadvantage acted as the second most common cause of conversion. In addition, frequency manipulation could change the preload and made the left ventricles' pre-existing diastolic dysfunction more sensitive to compromises. Hence, the heart's size was also an important consideration for OPBH CABG in our study, which was consistent with some studies.

The urgent switch rate in our study was 7.35%. Low cardiac output, IABP support, AKI requiring dialysis, duration of mechanic ventilation, and ICU stay had significant differences in urgent switch patients compared with OPBH patients, which showed the disadvantage of urgent switch. Some surgeons would like to choose the CPB assist (elective conversions) if they found it was difficult to anastomose during OPCABG before revascularization. As a report showed, this elective conversion often happened in patients with enlarged left ventricle, which was not associated with poorer outcomes^[27]^. To contrast with emergency conversion, elective conversion occurred as a planned measure to prevent the hemodynamics of deterioration during OPCABG. OPBH CABG could be seen as a method of elective conversion. A meta-analysis showed that the mortality rate might be 12-fold less if early conversion (3.1%) was chosen over late conversion (34.5%). In addition, 5-fold less in mortality rate, if conversion was elective (6.1%) than an emergency (32.1%)^[28-30]^. If the emergency conversion happened due to cardiac arrest, hemodynamic compromise, ischemic episodes and hemorrhage, MI occurrence, stroke, need for IABP, and ventilation time would increase significantly^[31-33]^. Therefore, the OPBH technique was an effective method to avoid emergency conversion in high-risk patients.

Some reports^[27]^ claimed that OPCABG was effective in low EF patients, especially those with COPD, which could avoid the disadvantages caused by CPB and shorter ventilating time. But, concerning that COPD was also a risk factor for emergency conversion and if these low LVEF patients have experienced urgent conversion, the left ventricle's dysfunction would increase the respiratory complications. So, OPBH CABG might be a better choice in such patients and the ventilation time of both groups showed no significance in our study; maybe it would be relevant with the shorter CPB time in the OPBH group.

In our study, elective conversion during OPCABG after anastomosis was not included, but 5 patients undergoing urgent switching during off-pump operation were included in the OPCABG group, which may have produced biased results. Three patients experienced ventricular fibrillation and 2 patients, hemodynamic deterioration, so urgent conversion was carried out. Among these 5 patients, 3 patients needed IABP support after operation, 1 patient died of low cardiac output and 1 patient died of malignant arrhythmia. During sudden ventricular fibrillation or hemodynamic deterioration, the visceral organs might experience short-time hypoperfusion injuries. Three patients needed dialysis due to AKI, which may be related to hypoperfusion perfusion. The short-time outcomes in urgent conversion were also worse in the OPCABG group than in the OPBH group. Thus, this result suggested that OPBH CABG maybe safer and act as a suitable choice for some moderate-risk patients with unstable hemodynamics during the operation.

This study presents several limitations. Firstly, it is a retrospective observational study with a single-center, small sample size that may influence the generalizability of the results. So, a final determination would need a prospective, multi-center study, with larger sample size. Secondly, the mean of the patients' age in this study was younger than in the past study, which contained a few COPD patients; the disadvantage of CPB in these patients is not so severe in respiratory complications and may have produced considerable bias. Thirdly, although reports have proved that CPB will not influence kidney function in a short time, one cannot be sure that the AKI in the OPBH group was relevant to the CPB or the poor left ventricular EF. And finally, the long-term clinical outcomes, especially MACE, need to be observed.

## CONCLUSION

In summary, our study showed some advantages of OPBH CABG, such as a greater number of grafts and more improved LVEF, compared with OPCABG. However, a slightly more drainage at the first 12 hours was the main drawback with CPB. It is difficult to conclude that OPBH can act as a standard procedure for patients with moderate left ventricular dysfunction. But OPBH CABG act as a complete revascularization, which provided better mid-term MACE-free survival rate compared with OPCABG.

**Table:** 

Authors' roles & responsibilities	
WW	Design of the study and coordination and help to draft the manuscript; approved the final manuscript
YW	Design of the study and coordination and help to draft the manuscript; approved the final manuscript
HP	Design of the study and coordination and help to draft the manuscript; approved the final manuscript
BL	Design of the study and coordination and help to draft the manuscript; approved the final manuscript
TW	Design of the study and coordination and help to draft the manuscript; approved the final manuscript
DL	Design of the study and coordination and help to draft the manuscript; approved the final manuscript
ZZ	Design of the study and coordination and help to draft the manuscript; approved the final manuscript
RX	Design of the study and coordination and help to draft the manuscript; approved the final manuscript
KL	Design of the study and coordination and help to draft the manuscript; approved the final manuscript
